# Oliguric acute kidney injury as a main symptom of bradycardia and arteriosclerosis resolved by pacemaker implantation: a case report

**DOI:** 10.1186/1752-1947-8-289

**Published:** 2014-09-01

**Authors:** Rainer U Pliquett, Daniel Radler, Alexander Tamm, Daniel Greinert, Robin Greinert, Matthias Girndt

**Affiliations:** 1 Department of Internal Medicine II, University Hospital Halle, Martin Luther University of Halle-Wittenberg, Halle, Saale, Germany; 2 Department of Internal Medicine III, Martin Luther University of Halle-Wittenberg, Halle, Saale, Germany; 3 Department of Internal Medicine I, Martin Luther University of Halle-Wittenberg, Halle, Saale, Germany

**Keywords:** Acute kidney injury, Atrioventricular block, Cardiorenal syndrome

## Abstract

**Introduction:**

Cardiovascular comorbidities regularly determine renal function. We report a case of acute kidney injury (Acute Kidney Injury Network stage 3) due to an intermittent third-degree atrioventricular block, which had not been diagnosed before.

**Case presentation:**

A 76-year-old Caucasian man with liver cirrhosis due to non-alcoholic fatty liver disease, and type-2 diabetes was cognitively impaired and had reduced vigilance presumably caused by hepatic encephalopathy and/or Alzheimer dementia. Within 2 years, two hospitalizations occurred for syncope attributed to orthostatic failure and hypovolemia. During the last hospitalization, oliguric acute kidney injury occurred. Sonography ruled out a post-renal cause. His renal resistive index was 1.0; his heart rate was below 50 beats per minute. After cessation of beta-blocker therapy, Holter electrocardiogram showed a new intermittent third-degree atrioventricular block with pauses for less than 3 seconds. Pacemaker insertion resolved his acute kidney injury, despite resumption of beta-blocker therapy. During four months of follow-up, syncope has not occurred, and vigilance was stable. However, his renal resistive index of 1.0 remained.

**Conclusions:**

Here, typical neurologic symptoms of bradycardia were misclassified. Diagnostic work-up of oliguric acute kidney injury revealed intermittent third-degree heart block. The pathomechanism of acute kidney injury relates to relevant bradycardia and increased vascular stiffness attenuating arterial diastolic renal blood flow.

## Introduction

Cardiovascular comorbidities regularly determine renal function and, vice versa, chronic renal insufficiency accelerates cardiovascular disease [1]. Hemodynamic instability or, in the worst case, hemodynamic shock is a common cause of oliguric acute kidney injury (AKI) and may be associated with arterial hypotension or arrhythmia, among others [2]. Clearly, heart rate within a certain range is a prerequisite for hemodynamic stability. As an example of hemodynamic instability, right-ventricular pacemaker stimulation of more than 200 beats per minute (bpm) is used to temporarily reduce cardiac output during percutaneous balloon aortic valvuloplasty, thus allowing for handling time [3].

Likewise, a relevant arrhythmia – either bradycardia or tachycardia – may reduce cardiac output temporarily, thereby attenuating kidney perfusion once renal autoregulation fails. A classification of cardiorenal syndrome covering five different scenarios has been proposed recently [4]. However, the detailed cause(s) and temporal interrelationship of combined cardiac and kidney failure often remain obscure. Alternatively, any concomitant renal and cardiac dysfunction may be regarded as cardiorenal syndrome, and immediate action is required when hemodynamic instability or shock has been reached.

For assessment of renal function, kidney ultrasound including Doppler sonography for determination of renal resistive index is mandatory. Renal resistive index is elevated when diastolic perfusion is absent or low. A diastolic no-flow condition due to reduced arterial Windkessel function may put individuals at risk for AKI during times of hemodynamic instability. For classification of AKI, the Acute Kidney Injury Network (AKIN) classification relies on the documented rise of serum creatinine over time and/or decrease of urine output [5]. Clearly, a reduced urine output is more sensitive for diagnosis of AKI than rise of serum creatinine [6,7]. In general, diagnostic measures of AKI aim to identify a possible renal stressor for timely treatment.

Here, we report a case of oliguric AKI (AKIN stage 3) due to intermittent bradycardia and attenuated diastolic kidney perfusion as reflected by a high renal resistive index which was resolved by pacemaker implantation.

## Case presentation

A 76-year-old man of Caucasian ethnicity was hospitalized for syncope 2 years before index hospitalization. Comorbidities included liver cirrhosis (Child-Pugh A) due to non-alcoholic fatty liver disease, type-2 diabetes, hypertension, and suspected Alzheimer-type dementia. Etiologically unclear transitory ischemic attacks had been diagnosed before. Holter electrocardiogram (ECG) showed a first-degree atrioventricular block and three episodes of bradycardia with minimal cardiac frequencies of 37bpm that were deemed unlikely as a cause for syncope. Schellong test revealed a hypodynamic cardiac response, that is, his heart rate stayed at 50bpm after change from supine to upright position. Hypoglycemia (under insulin therapy) was not detected prior, during or after hospitalization. A concomitant AKI (AKIN 2, maximum creatinine: 297μmol/L) was regarded as pre-renal, and syncope was attributed to arterial hypotension and hypovolemia. Following intravenous rehydration, he was discharged with spironolactone 150mg/day and propranolol 100mg/day for liver cirrhosis, donepezil 10mg/day for suspected dementia, amlodipine 10mg/day, and insulin subcutaneous injection (average: 50IU/day).

**Figure 1:**
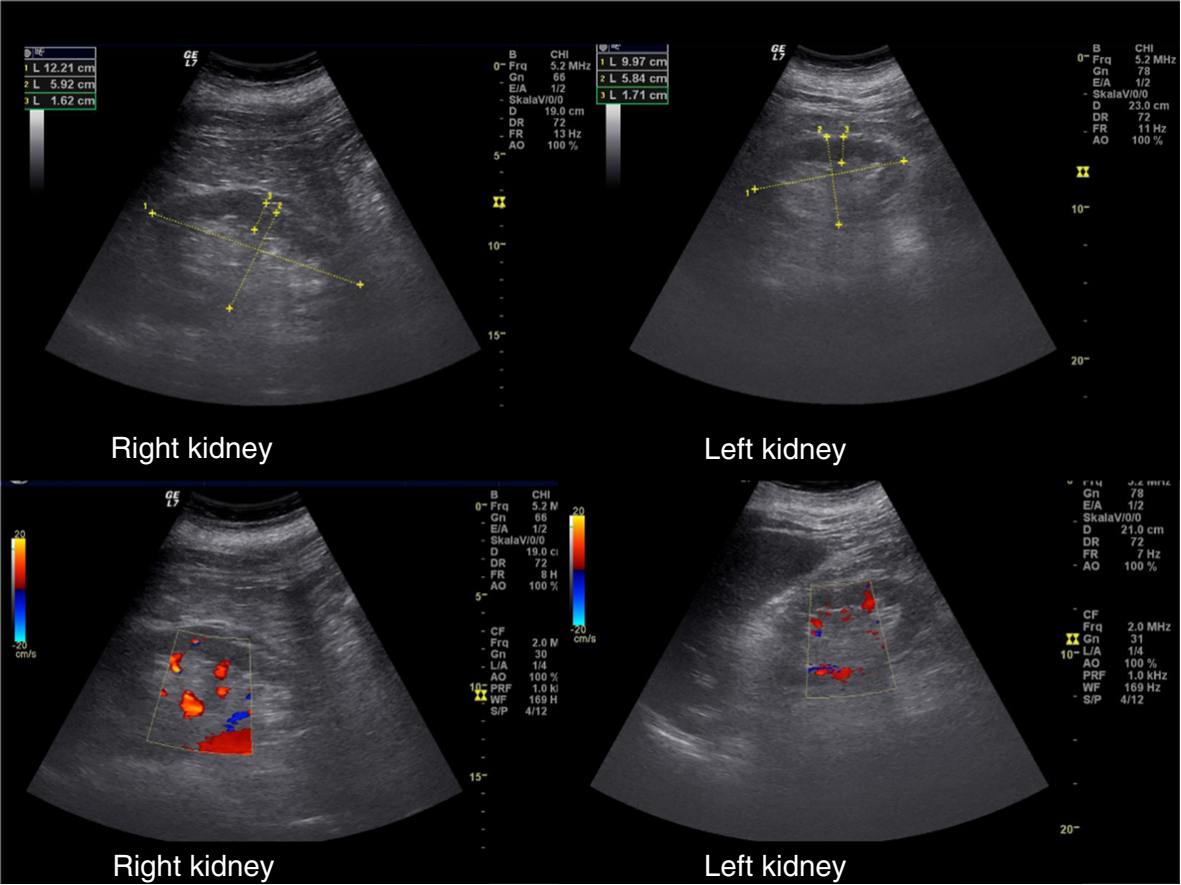
Ultrasound images obtained during index hospitalization showing nephrosclerotic kidneys with centralized arterial perfusion.

**Figure 2:**
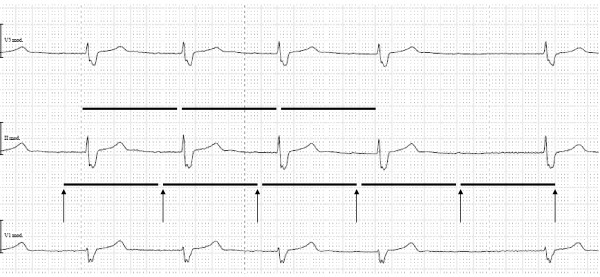
**Holter electrocardiogram reading of index hospitalization (paper speed 25mm/s) shows sinus rhythm with an average heart rate of 45 beats per minute, intermittent third-degree heart block, a pre-existing first-degree heart block and right-bundle branch block.** Arrowheads point to P waves. RR interval (bold upper line) is the same as the PP interval (bold lower line) indicating 1 to 1 conduction.

At index hospitalization, he (body mass index: 35.2) was admitted with nausea and vomiting for 3 days, as well as dizziness due to a presumed hepatic encephalopathy. On admission, vital parameters including blood pressure (150/80mmHg), heart rate (62bpm), and temperature and physical examination including cardiopulmonary state were unrevealing. Specifically, ascites and peripheral edema were absent. He was oriented to person, place, and time. Physical reactions were slowed though. Compared to baseline values prior to hospitalization (serum creatinine: 139μmol/L, estimated glomerular filtration rate [8]: 43.2μmol/L/1.73m^2^), serum creatinine was elevated on admission (Table 1). Glutamate-oxaloacetate transaminase (0.64), gamma-glutamyltransferase (2.46), and international normalized ratio (1.13) were slightly elevated; serum albumin (31g/L) was decreased. Other laboratory values including inflammation parameters (leukocyte count, C-reactive protein), total serum protein, bilirubin, glutamate-pyruvate transaminase, and alkaline phosphatase were within normal range. Partial respiratory insufficiency and metabolic acidosis (pH: 7.326, oxygen partial pressure: 55.8mmHg, carbon dioxide partial pressure: 29.0mmHg, bicarbonate: 14.7mmol/L, base excess: -9.7) were found on admission. His urine test showed microalbuminuria (<300mg/day), microscopic hematuria, and leukocyturia consistent with urinary tract infection. Concomitant medication largely remained unchanged since discharge 2 years ago. ECG showing sinus rhythm and the known first-degree atrioventricular block was without change since the last ambulatory cardiology check 1 year before. During hospitalization, he was bed-bound. Gastroscopy proved helicobacter-negative gastritis. An intravenous fluid challenge using sterile saline was performed for suspected, however clinically inapparent, hypovolemia. Despite fluid challenge, oliguria suddenly developed shortly after admission, serum creatinine remained high (Table 1). A pre- and post-renal cause of AKI was excluded. Myocardial infarction as a possible cause of hemodynamic instability was ruled out in repeat laboratory and ECG examinations. Echocardiography showed a normal systolic left-ventricular function, yet left-ventricular hypertrophy and diastolic dysfunction accompanied by a mild mitral regurgitation and an enlarged left atrium. Nephrotoxins and/or nephrotoxic medications were not applied. Euglycemia was verified during the whole index hospitalization. Kidney sonography revealed normal-sized kidneys with a centralized arterial perfusion (Figure 1), and an increased renal resistive index (1.0 on both sides) reflecting a diastolic no-flow condition. Of importance, a bradycardia (less than 50bpm) was present during ultrasound. Propranolol was discontinued at once, thereafter urine output improved (Table 1). In a Holter ECG 3 days following propranolol cessation, an intermittent third-degree atrioventricular block with pauses for less than 3 seconds was found (Figure 2). He was subjected to pacemaker insertion on day 11 after admission. Thereafter, renal function in terms of urine output and serum creatinine remained stable. Beta-blocker therapy was reinstituted. By discharge to a rehabilitation facility 14 days after pacemaker insertion, renal function in terms of serum creatinine further improved (Table 1), body weight remained constant in comparison to admission, and peripheral edema was absent.

**Table 1:** Collecting urine (24 hours), serum creatinine, urea, creatinine clearance prior, during, and after index hospitalization, if data were available

**Day prior / after hospital admission**	**24 hours collecting urine (mL)**	**Creatinine (μmol/L)**	**Blood urea nitrogen (mmol/L)**	**Creatinine clearance (mL/minute)**	**Estimated glomerular filtration rate (modification of diet in renal disease)**	**Action taken**
-732	ND	139	5.4	ND	43.2	
1	ND	200	ND	ND	ND	Admission, 1L saline (0.9%) i.v.
2	ND	ND	ND	ND	ND	1L saline (0.9%) i.v.
3	200	194	8.7	ND	ND	1L saline (0.9%) i.v.
4	300	ND	ND	ND		1L saline (0.9%) i.v., stop propranolol
5	600	219	10.1	ND		1L saline (0.9%) i.v., stop propranolol
6	ND	ND	ND	ND		1L saline (0.9%) i.v., stop propranolol
7	1000	179	8.6	ND		1L saline (0.9%) i.v., stop propranolol
8	700	ND	ND	ND		Stop propranolol
9	700	ND	ND	ND		Stop propranolol
10	ND	ND	ND	ND		Stop propranolol
11	ND	ND	ND	ND		Stop propranolol, pacemaker insertion
12	1000	ND	ND	ND		
13	ND	185	7.4	38.6	30.8	
…						
25	1000	162	3.7	32.3	35.9	Discharge
…						
48	ND	141	4.6	ND	46.6	

Abbreviations: i.v., intravenous; ND, no data.

At discharge, spironolactone 50mg daily, propranolol 25mg thrice daily, amlodipine (5mg daily), donezepil (10mg daily), and antidiabetic medication including sitagliptin and insulin were maintained. Ramipril (5mg daily), torasemide (20mg daily), acetylsalicylic acid (100mg daily), simvastatin (20mg daily), and pantoprazole (20mg daily) were prescribed.

**Figure 3:**
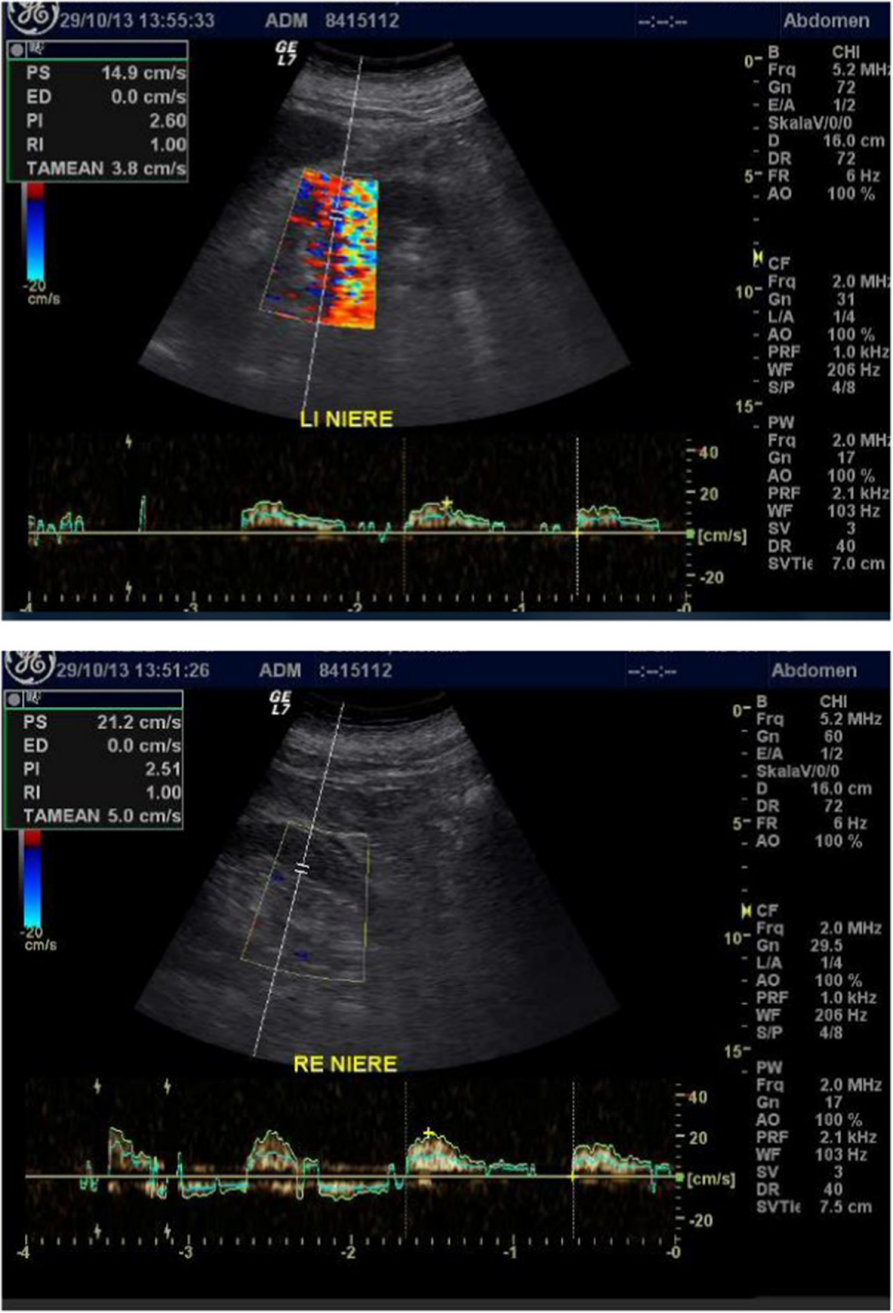
Follow-up kidney Doppler sonography examination at 4 months after discharge (upper panel: left side, lower panel: right side): resistive index of 1.0 on both sides.

Renal recovery continued after discharge, the prehospitalization level of creatinine was reached within 1 month following discharge.During the 4 months following discharge, amlodipine and ramipril were discontinued. Otherwise, medication has not changed. Syncope has not occurred again. His overall condition improved considerably. He was not bed-bound; he was alert and he could walk short distances with walking aid. Symptoms consistent with hepatic encephalopathy or progressive dementia were not present. Four weeks after discharge, his mini-mental state examination yielded 22 (out of 30) points. A neurologic reassessment was recommended regarding the ongoing donepezil treatment. At 4-months follow-up, sonography of his kidneys showed persistent renal resistive index of 1.0 on both sides (Figure 3).

## Conclusions

Here, bradycardia due to intermittent third-degree heart block led to an AKI stage 3 according to the urine-output criterion of the AKIN classification [5]. In fact, intravenous rehydration therapy did not improve oliguric AKI both in terms of diuresis or creatinine levels. However, cessation of beta-blocker treatment resolved oliguria. By discharge, antihypertensive medication was modified; that is, the dose of spironolactone and amlodipine was reduced, torasemide and the angiotensin-converting enzyme inhibitor ramipril were introduced. Lastly, pacemaker insertion led to a long-term recovery of AKI as judged on laboratory findings, diuresis and overall condition. Hypothetically, repeat AKI prior to hospitalization may explain his elevated creatinine on admission. For the AKI during index hospitalization, bradycardia appears to be the leading cause given the fact that pacemaker insertion stabilized kidney function, despite reinstitution of propranolol thereafter. Serum creatinine slowly decreased to prehospitalization levels within 1 month after discharge (Table 1), thus ruling out recurrent AKI after pacemaker insertion. As differential diagnosis, pre- or post-renal causes of AKI as well as infection or inflammatory disease were excluded in the present case. C-reactive protein was normal or only slightly elevated due to a urinary tract infection and gastritis.

The differential diagnosis of AKI further includes arterial hypotension which was not documented in this patient during index hospitalization. As a diagnostic dilemma, AKI due to hemodynamic instability may develop unnoticed when monitoring of heart rate and blood pressure are absent. Clues may emerge from follow-up diagnostics obtained after the fact. However, those diagnostic results need to be interpreted with caution. For instance, blood pressure may be elevated after onset of AKI due to renin-angiotensin-aldosterone system activation and/or hypervolemia as a consequence of oliguria. Thus, repeat measurements of blood pressure and Holter ECG are necessary to prove or exclude circulatory causes of recurrent AKI. In the present case, a likely circulatory cause of AKI remained obscure during the first hospitalization 2 years prior to index hospitalization. Arterial hypotension, although not documented in the present case, may have had a contributing role given the fact that ramipril and amlodipine have been stopped during follow-up.

Neither hepatic encephalopathy nor syncope occurred during 4 months following discharge. In retrospect, prior to pacemaker implantation, classic symptoms of (intermittent) third-degree atrioventricular block may have been obscured by immobilization or misinterpreted as hypovolemia or arterial hypotension. In addition, differential diagnosis included transitory ischemic attacks, Alzheimer type of dementia, and hepatic encephalopathy, all of which have to be questioned in retrospect. Specifically, liver function was stable during index hospitalization, ascites was absent, and symptoms attributed to hepatic encephalopathy were completely absent after pacemaker implantation. His Child-Pugh score on admission was only 4 points when discounting for hepatic encephalopathy.

Elevated renal resistive index signifies a loss of elastic vessel-wall properties or arteriosclerosis [9] leading to attenuated renal arterial perfusion during diastole. In the present case, halted renal arterial perfusion during diastole may be regarded as a precondition for AKI following periods of hemodynamic instability. Specifically, the combination of halted arterial kidney perfusion during diastole and bradycardia led to the AKI during hospitalization in terms of a mismatch between blood supply and demand.

In a prospective study on critically ill patients with sepsis, those experiencing AKI had a significantly higher renal resistive index than those without AKI [10]. It remains unclear whether renal resistive index was elevated in the acute situation or chronically. In the present case, renal resistive index was shown to be chronically elevated suggesting that arterial kidney perfusion is restricted to systole on a permanent basis. A meta-analysis of randomized clinical trials assessing antihypertensive medications in diabetics revealed that beta-blocker therapy confers no benefit prospectively, and possibly induces harm [11]. Hypothetically, underlying atherosclerosis hampering diastolic organ perfusion becomes clinically evident when using heart-rate lowering drugs.

This is the first one to the best of our knowledge where bradycardia and pre-existing low diastolic kidney perfusion were demonstrated to be the cause for oliguric AKI (AKIN stage 3). In fact, AKI was the starting point for diagnosis of third-degree atrioventricular block, even though 2 years elapsed from first symptoms until diagnosis. From the literature, an elderly patient with known chronic kidney disease (Kidney Disease: Improving Global Outcomes stage 3) and first-degree atrioventricular block coincidentally developed AKI (AKIN stage 3), hyperkalemia (5.8mmol/L) and third-degree heart block under diltiazem therapy (retarded release, 180mg/day) [12]. The authors concluded that diltiazem toxicity developed due to less renal elimination as a consequence of AKI leading to the third-degree heart block observed. Alternatively, AKI may be regarded as a consequence of third-degree atrioventricular block being an adverse effect of diltiazem in a patient with pre-existing first-degree heart block, the consequence of hyperkalemia, or the combination thereof. Clearly, the temporal relationship between those disorders remains unclear. However, a circulatory cause of AKI is a challenging differential diagnosis in this patient with bradycardia where information on renal-resistive index was not provided.

Taken together, both rhythmic instability in terms of bradycardia and attenuated diastolic arterial perfusion of the kidneys led to oliguric AKI (AKIN stage 3) in the present case. Classic signs and symptoms of bradycardia have been misclassified in the initially immobilized patient. Pacemaker implantation resolved this AKI. This case underlines the need to determine the role of chronically elevated renal resistive index for renal outcome in various clinical settings.

## Consent

Written informed consent was obtained from the patient for publication of this case report and any accompanying images. A copy of the written consent is available for review by the Editor-in-Chief of this journal.

## Competing interests

The authors declare that they have no competing interests.

## Authors’ contributions

RP conceived the case report and wrote the first version. DR, AT, DG, RG collected data and added valuable support. MG revised and approved the manuscript. All authors read and approved the final manuscript.
